# The susceptibility of *Aedes aegypti* populations displaying temephos resistance to *Bacillus thuringiensis israelensis*: a basis for management

**DOI:** 10.1186/1756-3305-6-297

**Published:** 2013-10-13

**Authors:** Ana Paula Araújo, Diego Felipe Araujo Diniz, Elisama Helvecio, Rosineide Arruda de Barros, Cláudia Maria Fontes de Oliveira, Constância Flávia Junqueira Ayres, Maria Alice Varjal de Melo-Santos, Lêda Narcisa Regis, Maria Helena Neves Lobo Silva-Filha

**Affiliations:** 1 Department of Entomology, Centro de Pesquisas Aggeu Magalhães-FIOCRUZ, Recife, PE 50670-420, Brazil

**Keywords:** Vector control, Bti, Susceptibility, Temephos, Metabolic resistance, Cross-resistance

## Abstract

**Background:**

*Aedes aegypti* is the vector of dengue virus, and its control is essential to prevent disease transmission. Among the agents available to control this species, biolarvicides based on *Bacillus thuringiensis* serovar *israelensis* (Bti) are an effective alternative to replace the organophosphate temephos for controlling populations that display resistance to this insecticide. The major goal of this study was to determine the baseline susceptibility of Brazilian *Ae*. *aegypti* populations to Bti, taking into account their background in terms of larvicide exposure, status of temephos resistance and the level of activity of detoxifying enzymes involved in metabolic resistance to insecticides.

**Methods:**

Population samples were established under insectarium conditions. Larval susceptibility to temephos and Bti was evaluated through bioassays and lethal concentrations of these compounds were determined. Biochemical assays were performed to determine the specific activity of five detoxifying enzymes in these samples.

**Results:**

Fourteen populations were characterized and, except for one case, all displayed resistance to temephos. Most populations were classified as highly resistant. The populations also showed increased activity of one or more detoxifying enzymes (glutathione-S-transferases, esterases and mixed function oxidases), regardless of their temephos resistance status. All populations analyzed were susceptible to Bti, and the lethal concentrations were similar to those detected in two laboratory susceptible colonies. The response to Bti showed little variation. A maximum resistance ratio of 2.1 was observed in two untreated populations, while in two Bti-treated populations, the maximum resistance ratio was 1.9. No positive correlation was found between temephos resistance, increased activity of detoxifying enzymes, and susceptibility to Bti.

**Conclusions:**

Data from this study show that all populations were susceptible to Bti, including twelve untreated and two treated populations that had been exposed to this agent for more than ten years. The temephos resistance and increased activity of detoxifying enzymes observed in thirteen populations was not correlated with changes in susceptibility to Bti. Our data show a lack of cross-resistance between these two compounds; thus, Bti can be used in an integrated control program to fight *Ae*. *aegypti* and counteract the temephos resistance that was found among all populations analyzed.

## Background

*Aedes aegypti* is the major vector of dengue virus (DENv) and has been responsible for an important disease burden in human populations worldwide in the last few decades [1,2]. This species has spread over most municipalities in Brazil, where it is the main DENv vector and has provoked frequent epidemics since 1986 [3]. Vector control remains the sole action to control dengue because vaccines and other prophylactic measures are not currently available. In this context, a National Program for *Aedes aegypti* Eradication (PEAa) was created in 1996 in Brazil and was replaced by the National Program for Dengue Control (PNCD) in 2002. The main goal of the PNCD is to fight this disease through integrated control actions, including the utilization of chemical larvicides and adulticides [4]. The organophosphate (OP) temephos has been the major larvicide used by the PNCD, and one of the most used compounds to control mosquitoes globally, despite its negative effects on non-target organisms and reports of resistance. In mosquito, temephos resistance has been associated with the alteration of its target site in acetylcholinesterase and also with metabolic mechanisms associated with enzymes involved in the detoxification of xenobiotic compounds [5-7].

Biolarvicides based on the entomopathogenic bacteria *Bacillus thuringiensis* serovar *israelensis* (Bti) have been successfully used for dipteran control [8]. Bti was first introduced for controlling *Simulium*, and its utilization was later extended to *Aedes* species. Long-term programs carried out in many countries have demonstrated its effectiveness under field conditions [9-11]. Its larvicidal action is based on crystals produced upon bacterial sporulation, mainly composed of the four protoxins Cry11Aa, Cry4Aa, Cry4Ba and Cyt1Aa. Bti’s mode of action depends on the ingestion of these crystals by larvae. Crystal solubilization occurs at the alkaline pH of the midgut, and the protoxins released into the lumen are converted into active toxins by proteases [12]. The whole crystal displays optimal toxicity, whereas individual toxins, or their combinations, do not show comparable levels of activity [13]. Once activated, the Cry toxins bind to specific midgut receptors from *Ae*. *aegypti* larvae: cadherins, aminopeptidases and alkaline phosphatases have been identified as binding molecules [14]. Studies to elucidate the synergy among Bti toxins have demonstrated that Cyt1Aa can act as a surrogate receptor for Cry11Aa and Cry4Ba. Furthermore, binding between Cyt1Aa and Cry toxins induces conformational changes that improve the capacity of Cry to bind to the other receptors available in the midgut [15-17]. This complex action based on four toxins with the capacity to bind to different target molecules does not favor the selection of resistance. Previous reports have failed to demonstrate the development of resistance to whole Bti crystal after continuous exposure for resistance selection under laboratory conditions [18-21], and resistance to Bti-based larvicides in field populations has not been reported to date [22,23].

In light of the effectiveness of Bti to control *Aedes* species and the lack of resistance, its utilization is under expansion in control programs for the treatment of breeding sites. Bti has also been used in oviposition and adult traps to prevent the development of larvae in these devices when they are used for monitoring, or on a massive scale, to reduce mosquito populations [24-26]. Another factor that supports the increasing use of Bti is the worldwide *Ae*. *aegypti* resistance to temephos, as has frequently been reported. In Brazil, there is a serious resistance problem that compromises the effects expected from use of temephos by the PNCD [27-33]. Thus, Bti is a candidate to manage resistance to temephos; however, some reports have recently suggested a potential cross-resistance between temephos and Bti [34-36]. These studies found a positive correlation between temephos resistance and increased activity of the detoxifying enzymes involved in the metabolism of xenobiotics along with a pattern of decreased response to Bti. This association was also reported for pyriproxyfen, suggesting that the increased activity of mixed function oxidases and previous temephos resistance could play a role in the reduced efficacy of pyriproxyfen against some species, including *Ae*. *aegypti*[37,38]. This issue requires investigation in view of the strategic role that Bti can play for the management of temephos resistance.

The major goal of the present study is to establish a baseline of Bti susceptibility in *Ae*. *aegypti* populations from Brazil, taking into account their background in terms of previous larvicide exposure, status of temephos resistance, and activity of detoxifying enzymes. Baseline data are necessary to evaluate the significance of alterations that can be found among populations exposed to control pressure and to provide guidelines for the use of control agents to be employed in mosquito control.

## Methods

### Colonies

Three *Aedes aegypti* colonies were used in this study and were maintained in the insectarium of the Centro de Pesquisas Aggeu Magalhães (CPqAM-FIOCRUZ) under controlled conditions at 26 ± 1°C, 70% humidity, and 12:12 h L:D photoperiod. Larvae were reared in dechlorinated tap water and fed with cat food. Adults were fed on a 10% sucrose solution, and females were also fed on chicken blood. The colonies used were: 1) Rockefeller, a susceptible colony used as an international reference for larvicides; 2) RecL, a susceptible colony established from a large egg sampling from the Recife Metropolitan Region (RMR) that has been maintained since 1996 in the insectarium of CPqAM-FIOCRUZ [39]; and 3) RecR, a temephos-resistant colony established from egg samples collected in Araripina city (Pernambuco State, Brazil). This sample showed an initial temephos resistance ratio (RR) of 7-fold, and further laboratory selection resulted in a RR at LC_95_ of 181-fold after 20 generations of temephos exposure [40,41].

### Populations

Mosquito samples from fourteen municipalities in Brazil collected between 2009 and 2011 were investigated in this study. Six were analyzed in the scope of the Brazilian *Aedes aegypti* Resistance Monitoring Network (MoReNAa), established by the PEAa in 2000. Ten samples were from different municipalities of Pernambuco State, while four (João Pessoa, Bacabal, Oiapoque and Macapá) were collected from other Brazilian States. All populations have been exposed to temephos except for the population from Fernando de Noronha, which is an oceanic island situated 354 km offshore of the Brazilian coast (Natal city). As this island is an Environmentally Protected Area, Bti-based products have been the sole larvicide used for *Aedes* control since 2002. In the other municipalities, the history of exposure to temephos, in the context of PEAa-PNCD actions, began in 1996. Recife city has the lowest level of exposure to temephos because this product was replaced by Bti in 2002. Insect growth regulators (IGR) acting as chitin synthesis inhibitors (Diflubenzuron or Novaluron) were introduced as a third control agent in two municipalities, Macapá and João Pessoa.

### Establishment of sub-colonies in the laboratory

Eggs from the municipalities analyzed were collected using oviposition traps set up in a representative number of sites according to the recommendations of the MoReNAa network [42]. Each population sample was established in the insectarium as a sub-colony composed of at least 1,000 adults obtained from the egg samples using a 2:1 female/male ratio. After hatching, larvae were reared until adulthood under the laboratory conditions previously described. Bioassays were carried out using larvae from the first (F_1_) or second (F_2_) generations obtained from these samples.

### Characterization of *Aedes aegypti* samples

Samples were analyzed according to the following parameters: 1) previous exposure to either the OP temephos, to the microbial agent Bti or to an IGR (Diflubenzuron or Novaluron); 2) status of temephos susceptibility; and 3) activity of detoxifying enzymes. The record of exposure to control agents was considered since 1996, when PEAa was implemented in Brazil. This information was provided by the Secretary of Health of the various municipalities.

### Temephos bioassays

Bioassays to evaluate temephos toxicity against larvae were performed according to a standard protocol [43]. Briefly, groups of 20 late 3^rd^ instar larvae in 100 mL of tap water in disposable cups were treated with a series of temephos concentrations that provide between 10 and 100% mortality after 24 h. Each bioassay was performed using six to ten concentrations and three replicates of 20 larvae per concentration, in addition to an untreated group. Mortality rates were recorded after 24 h to determine the lethal concentration for 50% (LC_50_) and 95% (LC_95_) of larvae using Probit analysis in the program SPSS 10.0 for Windows. The LCs established for each population were the average of at least three bioassays. The resistance ratios (RR) between the LC for the sample tested and the LC for the reference colony were used to classify the populations [44] into the following categories: low resistance (3 < RR< 5), moderate resistance (5 < RR < 10) and high resistance (RR > 10).

### Bti bioassays

Susceptibility to *Bacillus thuringiensis* serovar *israelensis* (Bti) was also analyzed through a similar protocol of multiple concentration bioassays using late 3^rd^ instar larvae, according to standard methods [45]. Lethal concentrations of the standard lyophilized powder of Bti strain H-14 (IPS82, Institut Pasteur) for 50% (LC_50_) and 90% (LC_90_) of exposed larvae after 24 h were determined. Briefly, groups of 20 larvae were exposed to serial dilutions of lyophilized spore-crystal standard powder in cups with 100 mL of bacterial suspensions in tap water. Three replicates were performed for each of six concentrations tested per bioassay. A control group was tested using water only. Each bioassay was repeated at least three times. The mean lethal concentrations and the resistance ratios (RR) were obtained for each sample, as described above.

### Enzymatic assays

The specific activity of detoxifying enzymes (DE), potentially associated with metabolic resistance to chemical insecticides, was evaluated. Three major classes of enzymes were assayed: glutathione-S-transferases (GST), esterases (α-est, ß-est, PNPA-est) and mixed function oxidases (MFO). For each population, approximately 100 one-day-old females, non-blood fed, previously stored at -70°C, were individually tested using a standard protocol described by Montella *et al*. (2007). The catalytic activity detected in the individuals from the Rockefeller colony was used as a reference to classify the tested samples according to the frequency of individuals that display an activity higher than the 99^th^ percentile of the Rockefeller population [46]. The samples were classified as unaltered (U <15%), altered (A 15-50%) and highly altered (HA >50%).

## Results

The study was performed based on sub-colonies of each population and three laboratory colonies, two used as references for susceptibility (Rockefeller and RecL) and one for temephos resistance (RecR), which was artificially selected in the laboratory. The evaluation of temephos toxicity to larvae from the sub-colonies showed that all populations analyzed were classified as resistant, except for the F. de Noronha population, which is from an area where temephos has not been used by the PEAa-PNCD (Table 1). Two populations (Bacabal and Recife) were classified as displaying moderate resistance (MR), while the remaining populations showed high resistance levels (HR) with a wide range of RR values, from 11-fold found in Macapá to 252.7 in Araripina. Among these HR populations, it was possible to distinguish two that displayed an RR of approximately 11-fold, where temephos was replaced by other control agents (Bti and IGR). A second group of nine populations that have been exposed exclusively to temephos since the beginning of the control program (Table 2), showed higher RR (60.0-252.7). Bacabal was the only population exposed exclusively to temephos that displayed a moderate level of resistance (RR 6.6-fold), rather than the high level of resistance that was observed for the other samples under this condition (Table 2). RR values for temephos resistance in half of the populations studied were over 100-fold.

**Table 1:** **Toxicity of temephos to 3rd instar *Aedes aegypti *larvae from colonies and sampled populations**

		**LC**_**50**_^**a**^		**LC**_**95**_^**a**^	
**Sample**	**No. larvae**	**Mean (95% fiducial limits)**	**RR**^**b**^	**Mean (95% fiducial limits)**	**RR**^**b**^
Rockefeller^c^	1200	0.007 (0.006-0.008)	1.0	0.011 (0.011-0.012)	1.0
RecL	1500	0.010 (0.009-0.010)	1.4	0.017 (0.016-0.019)	1.5
RecR (F_20_)^d^	1380	1.230 (1.168-1.305)	175.7	1.978 (1.840-2.180)	179.8
F. de Noronha	1740	0.017 (0.016-0.018)	2.4	0.026 (0.024-0.028)	2.4
Bacabal	2560	0.039 (0.037-0.043)	5.6	0.073 (0.069-0.079)	6.6
Macapá	1760	0.064 (0.057-0.070)	9.1	0.121 (0.103-0.137)	11.0
João Pessoa	1840	0.041 (0.033-0.043)	5.8	0.129 (0.103-0.173)	11.7
G. do Goitá	1560	0.135 (0.113-0.159)	19.3	0.792 (0.607-1.122)	72.0
Oiapoque	2240	0.295 (0.247-0.337)	42.1	1.127 (0.872-2.495)	102.5
Agrestina	1740	0.483 (0.421-0.548)	69.0	2.339 (1.899-3.051)	212.6
Araripina	1920	1.570 (1.420-1.750)	224.3	2.780 (2.600-3.280)	252.7
Rockefeller^c^	1140	0.012 (0.011-0.013)	1.0	0.017 (0.016-0.019)	1.0
Salgueiro	1500	0.154 (0.130-0.181)	12.8	1.021 (0.757-1.534)	60.0
S. C. Capibaribe	1320	0.675 (0.585-0.767)	56.2	2.421 (2.010-3.086)	142.4
S. J. Egito	1500	0.900 (0.818-0.986)	75.0	2.120 (1.862-2.514)	124.7
A. da Ingazeira	1260	1.013 (0.987-1.147)	84.4	2.051 (1.806-2.449)	120.6
Cedro	1320	1.063 (0.959-1.162)	88.6	2.256 (2.043-2.574)	132.7
Rockefeller^c^	1260	0.009 (0.009-0.010)	1.0	0.014 (0.014-0.016)	1.0
Recife	1680	0.042 (0.039-0.046)	4.7	0.100 (0.088-0.119)	7.1

^a^Lethal Concentrations (mg/L) for 50% (LC_50_) or for 95% (LC_95_) of exposed larvae after 24 h.

^b^Resistance Ratio (LC for population tested/LC for the Rockefeller reference colony).

^c^Data in this line are the reference (Rockefeller colony) for the samples below.

^d^Data from Strode *et al*. (2012).

**Table 2:** **Characterization of *Aedes aegypti *from laboratory colonies and sampled populations**

			**Temephos**		**Detoxifying enzymes**^**a**^				
**Sample**	**Origin**	**Exposure**^**b**^	**RR**_**95**_^**c**^	**Status**^**d**^	**GST**	**α-est**	**P-est**	**β-est**	**MFO**
Rockefeller	Laboratory	N	1.0	S	RF	RF	RF	RF	RF
RecL	Laboratory	N	1.0	S	U	U	U	U	U
RecR (F_20_)^e^	Laboratory	Temephos	179.8	HR	A	A	U	U	A
F. de Noronha	Field	Bti	2.4	S	HA	A	HA	U	U
Bacabal	Field	Temephos	6.6	MR	HA	A	A	U	A
Recife	Field	Temephos/Bti	7.1	MR	A	U	U	U	U
Macapá	Field	Temephos/Bti/IGR	11.0	HR	A	HA	HA	A	U
João Pessoa	Field	Temephos/Bti/IGR	11.7	HR	HA	A	HA	U	U
Salgueiro	Field	Temephos	60.0	HR	HA	U	HA	U	A
G. do Goitá	Field	Temephos	72.0	HR	A	A	A	U	U
Oiapoque	Field	Temephos	102.5	HR	A	HA	HA	U	U
Cedro	Field	Temephos	132.7	HR	HA	A	A	U	HA
A. da Ingazeira	Field	Temephos	120.6	HR	HA	HA	HA	A	U
S. J. do Egito	Field	Temephos	124.7	HR	HA	U	A	U	A
S. C. do Capibaribe	Field	Temephos	142.4	HR	HA	A	HA	U	U
Agrestina	Field	Temephos	212.6	HR	HA	A	A	U	A
Araripina	Field	Temephos	252.7	HR	A	HA	A	U	U

^a^GST (glutathione-S-transferases), α-est (α-esterases), P-est (PNPA esterases), β-est (β-esterases) and MFO (mixed-function oxidases) were classified according to Brasil (2006): RF-reference, U-unaltered, A-altered, HA-highly altered.

^b^Exposure records until 2010 for temephos, Bti-*Bacillus thuringiensis* serovar *israelensis*, IGR (Diflubenzuron or Novaluron).

^c^Resistance Ratio at Lethal Concentrations (mg/L) for 95% of exposed larvae after 24 h: LC for sample tested/LC for the Rockefeller colony.

^d^Classification adapted from Mazzari and Georghiou (1995): S-Susceptible (RR < 3), LR-Low Resistance (3 < RR < 5), MR-Moderate Resistance (5 < RR < 10), HR-High Resistance (>10).

^e^Data from Strode *et al*. (2012).

Individuals were then evaluated for the activity of DE. Thirteen populations showed increased or highly increased activity of at least three of the five enzymes investigated compared to the Rockefeller colony (Table 2, Additional file 1). The Recife population displayed an increase only in the activity of GSTs. In fact, GST activity was increased in all populations, followed by PNPA-esterases (93% of populations), α-esterases (79%), mixed function oxidases (36%) and finally β-esterases, whose alterations were less frequent amongst the populations (14%). Evaluation of laboratory colonies showed that RecL did not display any alterations, while RecR showed increased GST, α-esterases and MFO activities.

These analyses, summarized in Table 2, provide quantitative data on temephos resistance and DE activity in these populations as a basis for evaluating their susceptibility to Bti. The RecL reference colony and the temephos-resistant RecR colony were both susceptible to Bti, indicated by an RR that was equal or less than 2-fold at LC_50_ and LC_90_, compared to the Rockefeller colony (Table 3). The high level of temephos resistance and the increased activity of three groups of DE detected in the RecR colony were not correlated with a decrease in the susceptibility to Bti.

**Table 3:** **Toxicity of *Bacillus thuringiensis israelensis *(IPS82) to 3rd instar *Aedes aegypti *larvae from laboratory colonies**

					**LC**_**50**_^**a**^		**LC**_**90**_^**a**^	
**Samples**	**Origin**	**Temephos status**	**DE**^**c**^	**No. larvae**	**Mean (95% fiducial limits)**	**RR**^**b**^	**Mean (95% fiducial limits)**	**RR**^**b**^
Rockefeller	USA	Susceptible	RF	1620	0.008 (0.007-0.009)	1.0	0.026 (0.021-0.036)	1.0
RecL	Recife-PE	Susceptible	No	1020	0.016 (0.012-0.020)	2.0	0.030 (0.026-0.039)	1.2
RecR	Araripina-PE	Highly resistant	Yes	1620	0.010 (0.009-0.012)	1.3	0.030 (0.025-0.040)	1.2

^a^Lethal Concentrations (mg/L) for 50% (LC_50_) or 90% (LC_90_) of larvae after 24 h.

^b^Resistance Ratio: LC for sample tested/LC for the Rockefeller reference (RF) colony.

^c^Increase of the activity of detoxifying enzymes (DE).

The populations displaying different levels of temephos resistance were evaluated to provide a realistic measure of susceptibility to Bti. It should be noted that the F. de Noronha and Recife populations have been exposed to Bti, in particular the former, because this is the sole larvicide that has been used for mosquito control in that area. Toxicity assays showed that both were susceptible to Bti, with the RRs at LC_90_ similar to the reference colony (Table 4). The second group, Bacabal, Macapá and João Pessoa, which had temephos RRs between 6.6 and 11.7, was susceptible to Bti. The Bti RR’s were between 1.2 and 1.7 (Table 4). The last group analyzed, including populations characterized as highly resistant to temephos, presented RR values of between 60 and 252.7 (Table 1). However, all samples from this group were fully susceptible to Bti, with RR values at LC_90_ between 1.0- and 1.3-fold compared with the Rockefeller colony.

**Table 4:** **Toxicity of *Bacillus thuringiensis israelensis *(IPS82) to 3rd instar *Aedes aegypti *larvae from sampled populations**

				**LC**_**50**_^**a**^	**LC**_**90**_^**a**^		
**Samples**	**Origin**	**Temephos status**^**b**^	**No. larvae**	**Mean (95% fiducial limits)**	**RR**^**c**^	**Mean (95% fiducial limits)**	**RR**^**c**^
Rockefeller	Laboratory	S	1620	0.008 (0.007-0.009)	1.0	0.026 (0.021-0.036)	1.0
F. de Noronha	Field	S	1140	0.013 (0.011-0.015)	1.6	0.030 (0.024-0.042)	1.2
Bacabal	Field	MR	1140	0.014 (0.012-0.016)	1.8	0.043 (0.035-0.058)	1.7
Recife	Field	MR	1440	0.015 (0.014-0.018)	1.9	0.027 (0.024-0.031)	1.0
Macapá	Field	HR	1380	0.012 (0.011-0.014)	1.5	0.031 (0.027-0.038)	1.2
João Pessoa	Field	HR	1380	0.011 (0.009-0.012)	1.4	0.035 (0.027-0.049)	1.3
Salgueiro	Field	HR	1080	0.015 (0.012-0.018)	1.9	0.025 (0.021-0.031)	1.0
G. do Goitá	Field	HR	1080	0.015 (0.013-0.018)	1.9	0.025 (0.022-0.033)	1.0
Oiapoque	Field	HR	1440	0.011 (0.004-0.012)	1.4	0.026 (0.021-0.030)	1.0
Cedro	Field	HR	1200	0.012 (0.010-0.015)	1.5	0.031 (0.024-0.068)	1.2
A. da Ingazeira	Field	HR	1260	0.017 (0.016-0.020)	2.1	0.030 (0.026-0.035)	1.2
S. J. do Egito	Field	HR	1200	0.011 (0.010-0.013)	1.4	0.030 (0.024-0.038	1.2
S. C. do Capibaribe	Field	HR	1260	0.009 (0.008-0.010)	1.1	0.025 (0.018-0.051)	1.0
Agrestina	Field	HR	1320	0.017 (0.016-0.019)	2.1	0.028 (0.026-0.032)	1.1
Araripina	Field	HR	1440	0.013 (0.012-0.015)	1.6	0.035 (0.029-0.045)	1.3

^a^Lethal Concentrations (mg/L) for 50% (LC_50_) or 90% (LC_90_) of larvae after 24 h. ^b^Classification adapted from Mazzari and Georghiou (1995): S-Susceptible (RR < 3), LR-Low Resistance (3 < RR < 5), MR-Moderate Resistance (5 < RR < 10), HR-High Resistance (>10).

^c^Resistance Ratio: LC for sample tested/LC for the Rockefeller reference colony.

Analysis of Bti susceptibility in all samples showed a slight variation in the lethal concentrations, regardless of the other characteristics revealed by this study. Lethal concentrations at LC_50_ (mg/L) varied from 0.009 (S. C. do Capibaribe) to 0.017 (e.g., A. da Ingazeira), while at LC_90_, the values were between 0.025 (e.g., Salgueiro) and 0.043 (Bacabal). The slope of the dose–response curve for Bti assays performed in this study showed a homogenous response in the samples analyzed (data not shown). All populations, including the RecR colony exposed to temephos, displayed increased or highly increased DE activity. However, this parameter was not associated with the different levels of temephos resistance among populations. For instance, the Bti susceptibility of larvae from F. de Noronha and Araripina was similar, but they presented marked differences for temephos susceptibility (susceptible and HR, respectively). However, both populations showed similar profiles of increased DE (GST, α-esterases and PNPA-esterases). The only sample that showed unaltered levels of DE, similar to the Rockefeller colony, was the RecL colony, whose contact with xenobiotic compounds has been very limited, or absent, due to its maintenance under laboratory conditions.

## Discussion

The lack of data on mosquito susceptibility to candidate insecticides is a limiting factor for the success of control programs. These programs have often been implemented without information on the resistance selection risk posed by a given control agent in the field. For this reason, evaluations are often performed using the susceptibility of laboratory colonies as a reference, which do not necessarily reflect the natural variations that can be found among field populations, which has been demonstrated in other studies [47].

The results of this study provided a baseline dataset on the susceptibility of field populations of *Ae*. *aegypti* from Brazil to Bti. We also documented pre-existing exposure of the populations to control agents, in particular, to temephos, which has been largely used by the PNCD [4]. Indeed, thirteen of fourteen populations investigated displayed high resistance to temephos, and all displayed high activity of detoxifying enzymes, including GST and esterases, which could be a consequence of prolonged and intensive use of temephos. The resistance ratios (RR) found for these samples were much higher than those observed in previous surveys from Brazil, in which RRs were lower than 20 in several populations, except for two populations from Ceará and Pernambuco States with RRs > 100 [27-33,40,48].

All *Ae*. *aegypti* populations in our study, regardless of their resistance to temephos and increased activity of DEs, displayed a level of susceptibility to Bti similar to two reference colonies. RR values at LC_50_ or LC_90_ were equal to or lower than 2, which is not considered biologically significant for resistance. These RRs are likely due to natural variations in toxicity ratios rather than to resistance selection, as observed in the studies described below. For instance, surveys carried out on around fifty *Culex pipiens* populations with no history of Bti exposure showed that variation in the susceptibility to Bti ranged from RRs of 2 to 12.5 [49,50], while populations from three Bti-treated areas displayed RRs < 1 [51]. In China, five populations of *Anopheles sinensis* that were exposed to chemical and microbial larvicides showed RRs to Bti between 1.7 and 5.9, although pre-treatment data were not available and it was not possible to estimate pre existing variation [52]. Surveys based on thirty untreated and treated *Aedes* spp. populations showed a narrow range of RRs [35,53-58]. Among these cases, the maximum recorded RR value at LC_95_ was 4, detected in a Bti-treated population of *Ae*. *rusticus* from France, a Bti-treated population of *Ae*. *aegypti* from Malaysia [35,58], and an untreated population of *Aedes albopictus* from Malaysia [56]. These data suggest that the RR values were not related to previous Bti exposure. The present study showed discrete variations in Bti RR values in *Ae*. *aegypti* populations, including two Bti-treated populations whose RRs were similar to the untreated populations. The data suggest that the variation of *Ae*. *aegypti* susceptibility to Bti could be lower than that detected for *C*. *pipiens*. To date, the only report of a high RR described in a field population previously exposed to Bti was in two *C*. *pipiens* populations from New York State (USA) that displayed RRs of 14 and 41 [59]. However, data on the susceptibility of these populations before Bti treatment were not available, so there is no evidence that these high RRs were associated with exposure to Bti or if they were simply natural variations.

The analysis of Bti susceptibility in the populations in our study was performed in light of the disseminated temephos resistance reported for many areas of Brazil, which was confirmed by our data. In fact, Bti and temephos have distinct active principles, modes of action and target sites in insects, leading to the hypothesis that no cross-resistance is expected to occur. Data from the present study support this assertion since all populations showed a pattern of Bti susceptibility similar to the reference colonies, regardless of temephos resistance. Likewise, the RecR resistant colony, whose high resistance level (RR ≈ 180) was achieved under laboratory conditions [40], was still susceptible to Bti. As a consequence, the results from this work are not in agreement with previous reports whose findings suggest cross–effects of temephos and Bti, linked to DE alterations [34-36]. Boyer *et al*. (2007) observed an association between the exposure of *Ochlerotatus cataphylla* (Diptera: Culicidae) populations to Bti and temephos with a decrease in the sensitivity to these compounds and with an increase in the activity of GSTs and α-esterases. Those authors suggested that DEs may be involved in Bti detoxification [36]. Another report showed that *Ae*. *rusticus* populations from a Bti-treated area in France had Bti RRs (LC_50_) of 7, which correlated with a 3-fold increase in GST activity. However, biochemical evidence that Bti toxins could be detoxified by these enzymes was not provided [35]. In a general view, the association of DE in the resistance to insecticidal toxins from bacterial larvicides has not been related, while the alteration of target sites seems to play a major role in this process, besides others as the failure of protoxin processing and innate immune responses [12,60-62]. The only report concerning the role of esterases in the metabolism of insecticidal bacterial toxins involved the resistance of the lepidopteran *Helicoverpa armigera* to Cry1Ac from *Bacillus thuringiensis*[63]. In this case, esterases from a resistant strain were able to bind the Cry1Ac toxin, which could be the basis of resistance, although further investigation on this mechanism is needed [64]. Our study showed that all *Ae*. *aegypti* populations exhibited increased or highly increased DE activity for at least three groups of enzymes that we investigated. In particular, esterases can be responsible for the detoxification of a wide range of xenobiotics, including insecticidal compounds. Nevertheless, the susceptibility of our sampled larvae to Bti was similar to the reference colonies (Rockefeller and RecL), which did not display alterations in any of the five enzymes investigated. We did not find an association between the increased activity of detoxifying enzymes and a decrease in susceptibility to Bti.

## Conclusions

Fourteen *Ae. aegypti* samples from Brazilian populations were susceptible to Bti. Most populations analysed displayed high resistance to temephos, as well as increased levels of some detoxifying enzymes. However, these features were not associated with a decrease in their susceptibility to Bti. These findings strongly reinforce the importance of Bti as an effective tool for *Ae*. *aegypti* control, considering the increasing environmental concerns of people living in urban and rural environments, as well as the urgent need for larvicides able to overcome previous resistance to temephos. The insecticidal crystal of Bti has a unique composition of protoxins whose mode of action is distinct from neurotoxic or growth-regulating compounds used for mosquito control and thus has a low potential to display cross-resistance.

## Competing interests

The authors declare that they do not have competing interests.

## Authors’ contributions

APA, LNR, CFJA, CMFO, MAVMS and MHNLSF conceived the study, designed and analyzed the experimental data; APA, DFD, EH RAB performed the experiments; MHNLSF wrote the manuscript. All authors reviewed the paper and agreed with the final version.

## Supplementary Material

Additional file 1 **Activity of detoxifying enzymes in *Aedes aegypti *adults from sampled populations compared to the Rockefeller colony.** Click here for file
