# Supplementary material for: The susceptibility of Aedes aegypti populations displaying temephos resistance to Bacillus thuringiensis israelensis: a basis for management
Source: Parasit Vectors. 2013 Oct 13;6:297. doi: 10.1186/1756-3305-6-297 (PMC3852962; doi:10.1186/1756-3305-6-297)
Supplement: Additional file 1 — Activity of detoxifying enzymes in Aedes aegypti adults from sampled populations compared to the Rockefeller colony. [file 1756-3305-6-297-S1.pdf]

Additional File 1 Activity of detoxifying enzymes in *Aedes aegypti* adults from sampled populations compared to the Rockefeller colony

Sample	Glutathione S-transferase (GST) (mmol/mg ptn/min)				α-esterase (nmol/mg ptn/min)				β-esterase (nmol/mg ptn/min)				Esterases-PNPA (Δabs/mg ptn/min)				Mixed function oxidases (nmol/mg ptn/min)			
	N[a]	p99[b]	% >p99[c]	S[d]	N	p99	% >p99	S	N	p99	% >p99	S	N	p99	% >p99	S	N	p99	% >p99	S
Rockefeller	114	1.97	-	-	104	65.87	-	-	112	98.83	-	-	119	5.33	-	-	113	47.35	-	-
RecL	69	2.58	4	U	74	75.63	11	U	120	108.41	3	U	120	5.55	2	U	103	59.06	8	U
RecR (F20)[e]	115	3.49	26	A	116	116.81	47	A	113	114.94	4	U	100	6.36	6	U	117	63.27	34	A
Rockefeller	123	1.41	-	-	120	49.30	-	-	113	73.58	-	-	116	3.69	-	-	119	66.12	-	-
F. Noronha	115	2.97	69	HA	83	96.34	17	A	99	166.33	12	U	108	6.54	71	HA	118	70.52	3	U
Bacabal	110	2.59	73	HA	110	56.91	20	A	90	76.30	2	U	111	6.89	27	A	116	97.63	28	A
Recife	151	2.58	33	A	192	56.52	9	U	186	70.54	1	U	151	5.14	13	U	189	73.05	4	U
Macapá	117	2.60	38	A	119	77.84	62	HA	116	138.52	42	A	108	8.12	69	HA	101	75.47	7	U
J. Pessoa	115	3.29	76	HA	114	72.35	33	A	111	68.04	1	U	112	8.02	71	HA	74	61.79	0	U
Salgueiro	115	2.53	83	HA	115	67.97	11	U	112	71.08	2	U	114	8.81	82	HA	106	118.09	38	A
Agrestina	76	3.68	61	HA	145	63.35	25	A	151	73.93	1	U	94	7.20	37	A	138	85.48	20	A
G. Goitá	112	3.66	42	A	99	64.59	19	A	103	95.30	4	U	113	5.57	31	A	77	73.63	9	U
A. Ingazeira	150	2.65	55	HA	117	105.53	68	HA	118	94.67	27	A	154	7.34	62	HA	152	78.66	5	U
Oiapoque	97	2.93	42	A	111	77.20	58	HA	111	119.97	14	U	107	11.31	79	HA	114	79.65	8	U
Cedro	114	5.07	87	HA	116	80.44	41	A	116	91.99	6	U	114	7.43	48	A	117	111.01	54	HA
S.J. Egito	118	2.79	75	HA	110	62.10	11	U	116	82.60	3	U	105	5.26	21	A	107	199.73	22	A
S.C. Capibaribe	115	2.81	86	HA	118	69.56	31	A	111	76.71	3	U	114	9.12	87	HA	116	71.79	6	U
Araripina	80	2.22	29	A	115	100.17	59	HA	116	93.38	14	U	39	5.43	31	A	72	44.59	0	U

[a] Number of tested individuals.
[b] 99th Percentile for the populations.
[c] Percentual of individuals that display an activity higher than the 99th Percentile of the Rockefeller reference.
[d] Classification of enzyme activity (Brasil, 2006) compared to control (Rockefeller): unaltered (U); altered (A) and highly altered (HA).
[e] Data on this line are the reference for the samples below.
